# Supplementary material for: A Campylobacter integrative and conjugative element with a CRISPR-Cas9 system targeting competing plasmids: a history of plasmid warfare?
Source: Microb Genom. 2021 Nov 12;7(11):000729. doi: 10.1099/mgen.0.000729 (PMC8743540; doi:10.1099/mgen.0.000729)
Supplement: Supplementary material 1 [file mgen-7-0729-s001.pdf]

LEGENDS TO SUPPLEMENTARY INFORMATION

Figure S1. Alignment of the native *C. jejuni* and *C. coli* Cas9 proteins with the CampyICE1 Cas9 proteins from *C. jejuni* and *C. coli* clades. Part A shows an alignment of the *C. jejuni* chromosomal Cas9 protein with the CampyICE1 Cas9 from a *C. jejuni* strain, whereas Part B combines the different version from the *C. jejuni* and *C. coli* clades. Asterisks indicate identical residues, full stops and colon indicate conservative substitutions. Functional domains of Cas9 are indicated in yellow shading, while conserved amino acid residues are highlighted by underlined bold typeface [34].

Figure S2. Genetic structure of CampyICE1 and related mobile genetic elements (integrative conjugative elements) present in related *Campylobacter* species, presented as output of a comparison of Prokka-generated annotations [38] using Clinker [43].

Figure S3. Graphical representation of genetic variability of the mobile elements CJIE1, CJIE2, CJIE3 and CJIE4 from *C. jejuni* and *C. coli* genomes, presented as output of a comparison of Prokka-generated annotations [38] using Clinker [43].

Figure S4. Graphical representation of genetic variability of the plasmids pCC42, pTet and pVir from *C. jejuni* and *C. coli* genomes, presented as output of a comparison of Prokka-generated annotations [38] using Clinker [43].

Table S1. Overview of *C. jejuni* and *C. coli* genomes used in this study, with the Genbank or Campylobacter PubMLST accession numbers, metadata (source, MLST clonal complex, sequence type), and the presence/absence of the CJIE1-4 and CampyICE1 mobile elements and the pCC42, pTet and pVir plasmids. The position on the cgMLST tree used in Figures 3 and 4 is provided for

ordering purposes.

Table S2. Sequences of CampyICE1 spacers and variant families with their predicted targets, as identified using CRISPRTarget [42].

Table S3. Description of the three CampyICE1 spacer arrays per CampyICE1-positive *C. jejuni* or *C. coli* genome, and linkage to plasmid carriage.

Table S4. Distribution of CampyICE1 plasmid-specific CRISPR-spacers and pVir, pTet and pCC42 plasmids in CampyICE1-positive *C. jejuni* and *C. coli*.

van Vliet et al, Figure S1

Part A

```
                       RuvC I                                 R-rich region
Cj_Cas9       VARILAFDIGISSIGWAFSENDELKDCGVRIFTKVENPKTGESLALPRRLARSARKRLAR
Cj_ICE1_Cas9  -MKIIGFNLGIANIGWALRENDEIIDCGVRVFDIPENPKNGNSLALERRENKARMKIVKR
                :*:.*::**:.****: ****: *****:*   ****.*:**** **  ::  * : *

Cj_Cas9       RKARLNHLKHLIANEFKLNYEDYQSFDESLAKAYKGSLISPYELRFRALNELLSKQDFAR
Cj_ICE1_Cas9  KKARMLATKTFLKKELNVDLS---------KLFLIGSTQSIYELRTKALSSLISKEELSA
              :***:   * :: :*:::: .              **  * **** :**..*:**::::

Cj_Cas9       VILHIAKRRGYDDIKNSDDKEKGAILKAIKQNEEKLANYQSVGEYLYKEYFQKFKENSKE
Cj_ICE1_Cas9  IILHIAKHRGYDDSALKN--ENGTIIEALNKNKEAMLKFKSVGEYFYKNFVQ-----NKE
              :******:*****   .:  *:*:*::*:::*:* : :::*****:**::.*     .**

Cj_Cas9       FTNVRNKKESYERCIAQSFLKDELKLIFKKQREFGFSFSKKFEEEVLSVAFYKRALKDFS
Cj_ICE1_Cas9  VKKIRNTTEDYSNSVPRSLLKQELDLILDKQKELGLIKNADFKAKLFEIIFFKRPLKDFS
              ..::**..*.*...:.:*:**:**.**:.**:*:*:  . .*: :::.: *:**.*****

Cj_Cas9       HLVGNCSFFTDEKRAPKNSPLAFMFVALTRIINLLNNLKNTEGILYTKDDLNALLNEVLK
Cj_ICE1_Cas9  NKIGNCIFFENEKRAAKNTISACEFVALGKVVNLLKSIEKDIGIVYEKDSINEIMSIILD
              : :*** ** :****.**:  *  **** :::***:.:.:  **:* **.:* ::. :*.

Cj_Cas9       NGTLTYKQTKKLLGLSDDYEFK-------GEKGTYFIEFKKYKEFIKALGE--HNLSQDD
Cj_ICE1_Cas9  KTSISYKKIRDILNLPQDINFKGLDYSKNNAENSKLVDLKKLNEFKKALGDGFTNLDKDI
              : :::**: :.:*.*.:* :**       . :.: ::::** :** ****:   **.:* 

Cj_Cas9       LNEIAKDITLIKDEIKLKKALAKY-DLNQNQIDSLSKLEFKDHLNISFKALKLVTPLMLE
Cj_ICE1_Cas9  LDSIATDITLTKDTATLKEKLKNYNVLNAEQIEKLSELVFNDHINLSLKALKQIIPLMYE
              *:.**.**** **  .**: * :*  ** :**:.**:* *:**:*:*:**** : *** *

Cj_Cas9       GKKYDEACNELNLKVAINEDKKDFLPAFNETYYKDEVTNPVVLRAIKEYRKVLNALLKKY
Cj_ICE1_Cas9  GKRYDEACELCNFTIAKNQEKSEYLPLFEKTRFAKDISSPVVIRAICEFRKLLNDIIRRY
              **:*****:  *:.:* *::*.::** *::* : .:::.***:*** *:**:** ::::*

                   RuvC II
Cj_Cas9       GKVHKINIELAREVGKNHSQRAKIEKEQNENYKAKKDAELECEKLGLKINSKNILKLRLF
Cj_ICE1_Cas9  GSVHKIHLELTRDFGISFSDRKKIIKEIEQNEQSRIKALETIKELKLEETSKNIQIVRLF
              *.****::**:*:.* ..*:* ** ** ::* ::: .*    ::* *: .****  :***

                                               HNH domain
Cj_Cas9       KEQKEFCAYSGEKIKISDLQDEKMLEIDHIYPYSRSFDDSYMNKVLVFTKQNQEKLNQTP
Cj_ICE1_Cas9  EDQKGICPYSGLKMDLNRLDE---LVIDYIRPYNRSLDDSYSNKILTFKKLSDLKQGKTP
              ::** :*.*** *:.:. *::   * **:* **.**:**** **:*.*.* .: * .:**

Cj_Cas9       FEAFGNDSAKWQKIEVLAKNLPTKKQKRILDKNYKDKEQKNFKDRNLNDTRYIARLVLNY
Cj_ICE1_Cas9  FEAFGEDEKLWAEINERIKEYNGKKRFKIFDKFFKDKKPFDFTEQTLQDTRWLTKLVASY
              *****:*.  * :*:   *:   **: :*:** :***:  :*.::.*:***::.:** .*

                                                                  RuvC III
Cj_Cas9       TKDYLDFLPLSDDENTKLNDTQKGSKVHVEAKSGMLTSALRHTWGFSAKDRNNHLHHAID
Cj_ICE1_Cas9  LNEYLSFLPISEDENTALGYGEKGSKQHVVLSSGMITQMLRNFWYLGFKNHKDYKNNAMD
               ::**.***:*:**** *.  :**** **  .***:*  **: * :. *::::: ::*:*

Cj_Cas9       AVIIAYANNSIVKAFSDFKKEQESNSAELYAKKISELDYKNKRKFFEPFSGFRQKVLDKI
Cj_ICE1_Cas9  AIIVAFTTNSIIFAFNNFKKELDLAKAEFYANKISESDYLLKRKFLPPFSGFKEQALEKV
              *:*:*:..***: **.:**** :  .**:**:**** **  ****: *****:::.*:*:

Cj_Cas9       DEIFVSKPERKKPSGALHEETFRKEEEFYQSYGGKEGVLKALELGKIRKVNGKIVK--NG
Cj_ICE1_Cas9  KNIFVSHSLKIKNKGTLHELTPLKIKELKNTYGDLD---LAVKLGKIRKYNDKYYANANG
              .:****:. : * .*.*** *  * :*: :.**. :    *::****** *.*     **
```

```
Cj_Cas9         DMFRVDIFKHKKTNKFYAVPIYTMDFALKVLPNKAVARSKKGEIKDWILMDENYEFCFSL
Cj_ICE1_Cas9    SLVRADLFVDKK-NKFHAVSIYKADFSTKKLPNKTPATTSNGETKEGIEMNENYNFCMSL
                .:.*.*:* .** ***:**.**. **: * ****: * :.:** *: * *:***:**:**

Cj_Cas9         YKDSLILIQTKDMQEPEFVYYNAFTSSTVSLIVSKHDNKFETLSKNQKILFKNANEKEVI
Cj_ICE1_Cas9    YKNTPISVKIKGMKEPIICYYHGFNTSGSKITYKKHDNNYHNLSEDEMVVFR-KNDK---
                **:: * :: *.*:** : **:.*.:*  .:  .****::..**::: ::*:  *:*

Cj_Cas9         AKSIGIQNLKVFEKYIVSALGEVTKAEFRQREDFKK
Cj_ICE1_Cas9    -ESIAVGKILEIKKYSISPSGELSLIENEERKWF--
                 :**.: ::  ::** :*. **::  * .:*: *
```

Part B

```
                                      RuvC I
C_jejuni_Cas9                 VARILAFDIGISSIGWAFSENDELKDCGVRIFTKVENPKTGESLALPRRL
C_coli_Clade1a_Cas9           MARILAFDIGISSIGWAFSENDELKDCGVRIFTKAENPKTGESLALPRRL
C_coli_Clade1b_Cas9           MARILAFDIGISSIGWAFSENDELKDCGVRIFTKAENPKTGESLALPRRL
C_coli_Clade1c_Cas9           -MKILGFDIGINSIGWAFVEDNQLQDCGVRLFTKAEDPKTKESLALPRRN
C_coli_Clade2_Cas9            -MKILGFDIGINSIGWAFVEDNQLQDCGVRLFTKAEDPKRKESLALPRRN
C_coli_Clade3_Cas9            -MKILGFDIGINSIGWAFVEDNQLQDCGVRLFTKAEDPKTRESLALPRRN
C_jejuni_ICE1_Cas9            -MKIIGFNLGIANIGWALRENDEIIDCGVRVFDIPENPKNGNSLALERRE
C_coli_ICE1_Clade1a_Cas9      -MKIIGFNLGIANIGWALRENDEIIDCGVRVFDIPENPKNGNSLALERRE
C_coli_ICE1_Clade1c_Cas9      -MKILGFNLGIANIGWALRVNDEIIDCGVRVFDIPENPKNGNSLALERRE
C_coli_ICE1_Clade2_Cas9       -MKILGFNLGIANIGWALRENDEIIDCGVRVFDIPENPKNGNSLALERRE
C_coli_ICE1_Clade3_Cas9       -MKILGFNLGIANIGWALRVNDEIIDCGVRVFDIPENPKNGNSLALERRE
                                :*:.*::** .****:  :::: *****:*   *:**  :**** **

                               R-rich region
C_jejuni_Cas9                 ARSARKRLARRKARLNHLKHLIANEFKLNYEDYQSFDESLAKAYKGSLIS
C_coli_Clade1a_Cas9           ARSARKRLARRKARLNHLKHLIANEFKLNYEDYQSFDESLAKAYKGSLIS
C_coli_Clade1b_Cas9           ARSARKRLARRKARLNHLKHLIANEFKLNYEDYQSFDESLAKAYKGSLIS
C_coli_Clade1c_Cas9           ARSNRRRLGRRRSRLIALKHIISKELKLNYQDYMANDRELPKAYEGRLIS
C_coli_Clade2_Cas9            ARSNRRRLGRRRSRLIALKHIISKELKLNYQDYIANDGELPKAYEGRLVS
C_coli_Clade3_Cas9            ARSNRRRLGRRRSRLIALKHIISKELKLNYQDYIANDGELPKAYEGRLVS
C_jejuni_ICE1_Cas9            NKARMKIVKRKKARMLATKTFLKKELNVDLSKLFLIGS---------TQS
C_coli_ICE1_Clade1a_Cas9      NKARMKIVKRKKARMLATKTFLKKELNVDLSKLFLIGS---------TQS
C_coli_ICE1_Clade1c_Cas9      NKARMKTIKRKKARMLTTKTFLKKEFGIDLSKLFLIGS---------TQS
C_coli_ICE1_Clade2_Cas9       NKARMKTIKRKKARMLTTKTFLKKEFGIDLSKLFLIGS---------TQS
C_coli_ICE1_Clade3_Cas9       NKARMKTIKRKKARMLTTKTFLKKEFGIDLSKLFLIGS---------TQS
                               ::  : : *:::*:   * :: :*: :: ..    .            *

C_jejuni_Cas9                 PYELRFRALNELLSKQDFARVILHIAKRRGYDD--IKNSDDKEKGAILKA
C_coli_Clade1a_Cas9           PYELRFRALNELLSKQDFARVILHIAKRRGYDD--IKNNGDEEKSEILKA
C_coli_Clade1b_Cas9           PYELRFRALNELLSKQDFARVILHIAKRRGYDD--IKNSDDKEKGAILKA
C_coli_Clade1c_Cas9           PYELRYKALNEKIEPKDLARVILHIAKHRGYMNKNEKKSNDNEKGKILSA
C_coli_Clade2_Cas9            PYELRYKALNEKIEPKDLARVILHIAKHRGYMNKNEKKSSDNEKGKILSA
C_coli_Clade3_Cas9            PYELRYKALNEKIEPKDLARVILHIAKHRGYMNKNEKKSSDNEKGKILSA
C_jejuni_ICE1_Cas9            IYELRTKALSSLISKEELSAIILHIAKHRGYDDSALKN----ENGTIIEA
C_coli_ICE1_Clade1a_Cas9      IYELRTKALSSLISKEELSAIILHIAKHRGYDDSALKN----ENGTIIEA
C_coli_ICE1_Clade1c_Cas9      IYELRTKALNSLISKEELAAIILHIAKHRGYDDSALKN----ENGIIIEA
C_coli_ICE1_Clade2_Cas9       IYELRTKALNSLISKEELAAIILHIAKHRGYDDSALKN----ENGIIIEA
C_coli_ICE1_Clade3_Cas9       IYELRTKALNSLISKEELAAIILHIAKHRGYDDSALKN----ENGIIIEA
                               **** :**.. :. :::: :******:*** :   *:    *:. *:.*

C_jejuni_Cas9                 IKQNEEKLANYQSVGEYLYKEYFQKFKENSKEFTNVRNKKESYERCIAQS
C_coli_Clade1a_Cas9           IKQNEEKLVNYQSVGEYLYKEYFQKFKENSKEFINVRNKKESYERCIAQS
C_coli_Clade1b_Cas9           IKQNEEKLANYQSVGEYLYKEYFQKFKENSKEFTNVRNKKESYERCIAQS
C_coli_Clade1c_Cas9           LKTNALKLEKYQSVGEYFYKEFFQKHKENTKDFINIRNKKGSYDNCVLAS
C_coli_Clade2_Cas9            LKTNALKLEKYQSVGEYFYKEFFQKYKENTKDFINIRNKEGSYENCVLAS
C_coli_Clade3_Cas9            LKTNALKLEKYQSVGEYFYKEFFQKYRENTKDFINIRNKEGSYENCVLAS
C_jejuni_ICE1_Cas9            LNKNKEAMLKFKSVGEYFYKNFVQN-----KEVKKIRNTTEDYSNSVPRS
C_coli_ICE1_Clade1a_Cas9      LNKNKEAMLKFKSVGEYFYKNFVQN-----KEVKKIRNTTEDYSNSVPRS
C_coli_ICE1_Clade1c_Cas9      LNKNKEEMLKFKSVGEYFYKNFVQS-----KEVRKIRNTTEDYSNSIPRS
C_coli_ICE1_Clade2_Cas9       LNKNKEEMLKFKSVGEYFYKNFVQS-----KEVRKIRNTTEDYSNSIPRS
C_coli_ICE1_Clade3_Cas9       LNKNKEEMLKFKSVGEYFYKNFVQS-----KEVRKIRNTTEDYSNSIPRS
                              :: *   : :::*****:**::.*.     *:. ::**.  .*...:  *
```

```
C_jejuni_Cas9                FLKDELKLIFKKQREFGFSFSKKFEEEVLSVAFYKRALKDFSHLVGNCSF
C_coli_Clade1a_Cas9          FLKDELKLIFQKQREFGFSFSKKFEEEVLSVAFYKRALKDFSHLVGNCSF
C_coli_Clade1b_Cas9          FLKDELKLIFKKQREFGFSFSKKFEEEVLSVAFYKRALKDFSHLVGNCSF
C_coli_Clade1c_Cas9          DLEKELKLILEKQKEWGYSYDDNFIKEILKVAFFQRPLKDFSYLVGVCTF
C_coli_Clade2_Cas9           DLEKELKLILEKQKEWGYSYNDNFIKEILKVAFFQRPLKDFSYLVGACTF
C_coli_Clade3_Cas9           DLEKELKLILEKQKEWGYSYNDNFIKEILKVAFFQRPLKDFSYLVGACTF
C_jejuni_ICE1_Cas9           LLKQELDLILDKQKELGLIKNADFKAKLFEIIFFKRPLKDFSNKIGNCIF
C_coli_ICE1_Clade1a_Cas9     LLKQELDLILDKQKELGLIKNADFKEKLFEIIFFKRPLKDFSNKIGNCIF
C_coli_ICE1_Clade1c_Cas9     LLKQELDLILNKQKELGLIKNADFKEKLFEIIFFKRPLKDFSNKIGNCIF
C_coli_ICE1_Clade2_Cas9      LLKQELDLILNKQKELGLIKNADFKEKLFEIIFFKRPLKDFSNKIGNCIF
C_coli_ICE1_Clade3_Cas9      LLKQELDLILNKQKELGLIKNADFKEKLFEIIFFKRPLKDFSNKIGNCIF
                              *:.**.**:.**:* *   . .*  :::.: *::*.*****  :* * *

C_jejuni_Cas9                FTDEKRAPKNSPLAFMFVALTRIINLLNNLKNTEGILYTKDDLNALLNEV
C_coli_Clade1a_Cas9          FTDEKRAPKNSPLAFMFVALTRIINLLNNLKNTEGILYTKDDLNALLNEV
C_coli_Clade1b_Cas9          FTDEKRAPKNSPLAFMFVALTRIINLLNNLKNTEGILYTKDDLNALLNEV
C_coli_Clade1c_Cas9          FEDEKRACKNSYSAWEFVALTKIINELKSLEKESGELVSSQIINEILNHV
C_coli_Clade2_Cas9           FEDEKRACKNSYSAWEFVALTKIINELKSLEKESGELVSSQIINEILNHI
C_coli_Clade3_Cas9           FEDEKRACKNSYSAWEFVALTKIINELKSLEKESGELVSSQIINEILNHI
C_jejuni_ICE1_Cas9           FENEKRAAKNTISACEFVALGKVVNLLKSIEKDIGIVYEKDSINEIMSII
C_coli_ICE1_Clade1a_Cas9     FENEKRAAKNTISACEFVALGKVVNLLKSIEKDIGIVYEKDSINEIMSII
C_coli_ICE1_Clade1c_Cas9     FENEKRAAKNTLSACEFVALGKVINLLKSIEKDTGIVYEKDIISEIMNII
C_coli_ICE1_Clade2_Cas9      FENEKRAAKNTLSACEFVALGKVINLLKSIEKDTGIVYEKDIISEIMNII
C_coli_ICE1_Clade3_Cas9      FENEKRAAKNTLSACEFVALGKVINLLKSIEKDTGIVYEKDIISEIMNII
                             * :**** **:  *  **** :::* *:.:::  * :  .: :. ::. :

C_jejuni_Cas9                LKNGTLTYKQTKKLLGLSDDYEFKG-------EKGTYFIEFKKYKEFIKA
C_coli_Clade1a_Cas9          LKNGTLTYKQTKKLLGLSDDYEFKG-------EKGTYFIEFKKYKEFIKA
C_coli_Clade1b_Cas9          LKNGTLTYKQTKKLLGLSDDYEFKG-------EKGTYFIEFKKYKEFIKA
C_coli_Clade1c_Cas9          LDKGSITYKKFREYIKLHESMKFKSLKYDKDNAESTKLIEFRKLVEFKKA
C_coli_Clade2_Cas9           LDKGSITYKKFREYIKLHESMKFKSLKYDKDNAESVKLIEFRKLVEFKKA
C_coli_Clade3_Cas9           LDKGSITYKKFREYIKLHESMKFKSLKYDKDNVESTKLIEFRKLVEFKKA
C_jejuni_ICE1_Cas9           LDKTSISYKKIRDILNLPQDINFKGLDYSKNNAENSKLVDLKKLNEFKKA
C_coli_ICE1_Clade1a_Cas9     LDKTSISYKKIRDILNLPQDINFKGLDYSKNNAENSKLVDLKKLNEFKKA
C_coli_ICE1_Clade1c_Cas9     LNKASISYKKIRDILNLPQDISFKGLDYSKNNAENSKLVDFKKLNEFKKA
C_coli_ICE1_Clade2_Cas9      LNKASISYKKIRDILNLPQDISFKGLDYSKNNAENSKLVDFKKLNEFKKA
C_coli_ICE1_Clade3_Cas9      LNKASISYKKIRDILNLPQDISFKGLDYSKNNAENSKLVDFKKLNEFKKA
                             *.: :::**: :. : * :. .**.        :.  :::::*  ** **

C_jejuni_Cas9                LGEH--NLSQDDLNEIAKDITLIKDEIKLKKALAKY-DLNQNQIDSLSKL
C_coli_Clade1a_Cas9          LGEH--NLSQDDLNEIAKDITLIKDEIKLKKALAKY-DLNQNQIDSLSKL
C_coli_Clade1b_Cas9          LGDH--SLSQDDLNEIAKDITLIKDEIKLKKALAKY-DLNQNQIDSLSKL
C_coli_Clade1c_Cas9          LGEH--SLTREELDQIATYITLIKDNEKLKITLEKY-SLNNEQIKNLIEI
C_coli_Clade2_Cas9           LGEH--SLTREELDQIATYITLIKDNEKLKITLEKY-SLNNEQIKNLIEI
C_coli_Clade3_Cas9           LGEH--SLSREELDQIATYITLIKDNEKLKITLEKY-SLNNEQIKNLLEI
C_jejuni_ICE1_Cas9           LGDGFTNLDKDILDSIATDITLTKDTATLKEKLKNYNVLNAEQIEKLSEL
C_coli_ICE1_Clade1a_Cas9     LGDGFANLDKDILDSIATDITLTKDTATLKEKLKNYNVLNAEQIEKLSEL
C_coli_ICE1_Clade1c_Cas9     LDDSFVNLDKDILDSIATDITLTKDMTALKEKLESYNVLNKEQIEKLSEL
C_coli_ICE1_Clade2_Cas9      LDDSFVNLDKDILDSIATDITLTKDMTALKEKLESYNVLNKEQIEKLSEL
C_coli_ICE1_Clade3_Cas9      LDDSFVNLDKDILDSIATDITLTKDMTALKEKLESYNVLNKEQIEKLSEL
                             *.:   .* :: *:.**. *** **   **  * .*  ** :**..* ::

C_jejuni_Cas9                EFKDHLNISFKALKLVTPLMLEGKKYDEACNELNLKVAINEDKKDFLPAF
C_coli_Clade1a_Cas9          EFKDHLNISFKALKLVTPLMLEGKKYDEACNELNLKVAINEDKKDFLPAF
C_coli_Clade1b_Cas9          EFKDHLNISFKALKLITPLMLEGKKYDEACNELNLKVAINEDKKDFLPAF
C_coli_Clade1c_Cas9          DFNDHINLSFKALNLILPLMKEGKRYDEGCKLLNLKTKSNNQKFDFLPAF
C_coli_Clade2_Cas9           DFNDHINLSFKALNLILPLMKEGKRYDEACKLLNLKTKSNNQKFDFLPAF
C_coli_Clade3_Cas9           DFNDHINLSFKALNLILPLMKEGKRYDEACKLLNLKTKSNNQKFDFLPAF
C_jejuni_ICE1_Cas9           VFNDHINLSLKALKQIIPLMYEGKRYDEACELCNFTIAKNQEKSEYLPLF
C_coli_ICE1_Clade1a_Cas9     VFNDHINLSLKALKQIIPLMYEGKRYDEACELCNFTIAKNQEKSEYLPLF
C_coli_ICE1_Clade1c_Cas9     AFNDYINLSLKALKQIIPLMYEGKRYDEACKLCNFAIAKNQEKSEFLPLF
C_coli_ICE1_Clade2_Cas9      AFNDYINLSLKALKQIIPLMYEGKRYDEACKLCNFAIAKNQEKSEFLPLF
C_coli_ICE1_Clade3_Cas9      AFNDYINLSLKALKQIIPLMYEGKRYDEACKLCNFAIAKNQEKSEFLPLF
                              *:*::*:*:***: : *** ***:***.*:  *:    *::* ::** *
```

```
                                                                   RuvC II
C_jejuni_Cas9                 NETYYKDEVTNPVVLRAIKEYRKVLNALLKKYGKVHKINIELAREVGKNH
C_coli_Clade1a_Cas9           NETYYKDEVTNPVVLRAIKEYRKVLNALLKKYGKVHKINIELAREVGKNH
C_coli_Clade1b_Cas9           NETYYKDEVTNPVVLRAIKEYRKILNALLKKYGKVHKINIELAREVGKNH
C_coli_Clade1c_Cas9           CDSIFAQELTNPIVNRAISEYRKVLNALLKKYGKMHKIHIELARDIGLSK
C_coli_Clade2_Cas9            CDSIFAQELTNPIVNRAISEYRKVLNALLKKYGKMHKIHIELARDIGLSK
C_coli_Clade3_Cas9            CDSIFAQELTNPIVNRAISEYRKVLNALLKKYGKMHKIHIELARDIGLSK
C_jejuni_ICE1_Cas9            EKTRFAKDISSPVVIRAICEFRKLLNDIIRRYGSVHKIHLELTRDFGISF
C_coli_ICE1_Clade1a_Cas9      EKTRFAKDISSPVVIRAICEFRKLLNDIIRRYGSVHKIHLELTRDFGISF
C_coli_ICE1_Clade1c_Cas9      EKTRFAKDISSPVVIRAVCEFRKLLNDIIRRYGSVHKIHLELTRDFGISF
C_coli_ICE1_Clade2_Cas9       EKTRFAKDISSPVVIRAVCEFRKLLNDIIRRYGSVHKIHLELTRDFGISF
C_coli_ICE1_Clade3_Cas9       EKTRFAKDISSPVVIRAVCEFRKLLNDIIRRYGSVHKIHLELTRDFGISF
                               .: : .:::.*:* **: *:**:** ::::**.:***::**:*:.* .

C_jejuni_Cas9                 SQRAKIEKEQNENYKAKKDAELECEKLGLKINSKNILKLRLFKEQKEFCA
C_coli_Clade1a_Cas9           SQRAKIEKEQNENYKAKKDAELECEKLGLKINSKNILKLRLFKEQKEFCA
C_coli_Clade1b_Cas9           SQRAKIEKEQNENYKAKKDAEIECEKLGLKINSKNILKLRLFKEQKEFCA
C_coli_Clade1c_Cas9           KLRTKIEKEQKENYENNIWALNECENFGLKANTKNILKLKLWKEQKEFCI
C_coli_Clade2_Cas9            KLRTKIEKEQKENYENNIWALNECENFGLKANAKNILKLKLWKEQKEFCI
C_coli_Clade3_Cas9            KLRAKIEKEQKENYENNIWALNECENFGLKANAKNILKLKLWKEQKEFCI
C_jejuni_ICE1_Cas9            SDRKKIIKEIEQNEQSRIKALETIKELKLEETSKNIQIVRLFEDQKGICP
C_coli_ICE1_Clade1a_Cas9      NDRKKIIKEIEQNEQSRIKALETIKELKLEETSKNIQIVRLFEDQKGICP
C_coli_ICE1_Clade1c_Cas9      NDRRKIIKEIEQNEQSRIKALETIKELKLEETPKNIQIVRLFGDQKGICP
C_coli_ICE1_Clade2_Cas9       NDRRKIIKEIEQNEQSRIKALETIKELKLEETPKNIQIVRLFGDQKGICP
C_coli_ICE1_Clade3_Cas9       NDRRKIIKEIEQNEQSRIKALETIKELKLEETPKNIQIVRLFGDQKGICP
                              . * ** ** ::* : .  *    ::: *: ..***  ::*: :** :*

                                                        HNH domain
C_jejuni_Cas9                 YSGEKIKISDLQDEKMLEIDHIYPYSRSFDDSYMNKVLVFTKQNQEKLNQ
C_coli_Clade1a_Cas9           YSGEKIKISDLQDEKMLEIDHIYPYSRSFDDSYMNKVLVFTKQNQEKLNQ
C_coli_Clade1b_Cas9           YSGEKIKLSDLQDEKMLEIDHIYPYSRSFDDSYMNKVLVFTKQNQEKLNK
C_coli_Clade1c_Cas9           YSGKKISIEHLRDEKTLEVDHIYPYSRSFDDSFLNKVLVFTKENQEKLNK
C_coli_Clade2_Cas9            YSGKKISIEHLRDEKALEVDHIYPYSRSFDDSFLNKVLVFTKENQEKLNK
C_coli_Clade3_Cas9            YSGKKISIEHLRDEKALEVDHIYPYSRSFDDSFLNKVLVFTKENQEKLNK
C_jejuni_ICE1_Cas9            YSGLKMDLNRLDE---LVIDYIRPYNRSLDDSYSNKILTFKKLSDLKQGK
C_coli_ICE1_Clade1a_Cas9      YSGLKMDLKCLDE---LVIDYIRPYNRSLDDSYSNKVLTFKKLNDLKQGK
C_coli_ICE1_Clade1c_Cas9      YSGLKMDLNRLDE---LVIDYIRPYNRSFDDSYSNKVLTFKKLNDLKQGK
C_coli_ICE1_Clade2_Cas9       YSGLKMDLNRLDE---LVIDYIRPYNRSFDDSYSNKVLTFKKLNDLKQGK
C_coli_ICE1_Clade3_Cas9       YSGLKMDLNRLDE---LVIDYIRPYNRSFDDSYSNKVLTFKKLNDLKQGK
                              *** *:.:. * :   * :*:* **.**:***: **:*.*.* .: * .:

C_jejuni_Cas9                 TPFEAFGNDSAKWQKIEVLAKNLPTKKQKRILDKNYKDKEQKNFKDRNLN
C_coli_Clade1a_Cas9           TPFEAFGNDSAKWQKIEVLAKNLPTKKQKRILDKNYKDKEQKNFKDRNLN
C_coli_Clade1b_Cas9           TPFEAFGNDSTKWQKIEVLAKNLPEKKQKRILDKNYKDKEQKDFKDRNLN
C_coli_Clade1c_Cas9           TPFEAFGANEERWSKIQALAQNLPYKKKNKILDEAFKGKQQQDFISRNLN
C_coli_Clade2_Cas9            TPFEAFGANEERWSKIQALAQNLPYKKKNKILDEAFKGKQQQDFISRNLN
C_coli_Clade3_Cas9            TPFEAFGANEERWSKIQALAQNLPYKKKNKILDEAFKGKQQQDFISRNLN
C_jejuni_ICE1_Cas9            TPFEAFGEDEKLWAEINERIKEYNGKKRFKIFDKFFKDKKPFDFTEQTLQ
C_coli_ICE1_Clade1a_Cas9      TPFEAFGEDEKLWAEINERIKEYNGKKRFKIFDKFFKDKKPFDFTEQTLQ
C_coli_ICE1_Clade1c_Cas9      TPFEAFGEDEKLWAEINERIKEYNGKKRFKIFDKFFKDKKPFDFTEQTLQ
C_coli_ICE1_Clade2_Cas9       TPFEAFGEDEKLWAEINERIKEYNGKKRFKIFDKFFKDKKPFDFTEQTLQ
C_coli_ICE1_Clade3_Cas9       TPFEAFGEDEKLWAEINERIKEYNGKKRFKIFDKFFKDKKPFDFTEQTLQ
                              ******* :.  * :*:   ::   **: :*:*: :*.*:  :* .:.*:

C_jejuni_Cas9                 DTRYIARLVLNYTKDYLDFLPLSDDENTKLNDTQKGSKVHVEAKSGMLTS
C_coli_Clade1a_Cas9           DTRYIARLVLNYTKDYLDFLPLSDDENTKLNDTQKGSKVHVEAKSGMLTS
C_coli_Clade1b_Cas9           DTRYIARLVLNYTKDYLDFLPLSDDENTKLNDTQKGSKVHVEAKSGMLTS
C_coli_Clade1c_Cas9           DTRYISTLIVKYTKEYLDFLPLDEKEDISLKSGEKGSKIHVQTINGMLTS
C_coli_Clade2_Cas9            DTRYISTLIVKYTKEYLDFLPLDEKEDINLKSGEKGSKIHVQTINGMLTS
C_coli_Clade3_Cas9            DTRYISTLIVKYTKEYLDFLPLDEKEDINLKSGEKGSKIHVQTINGMLTS
C_jejuni_ICE1_Cas9            DTRWLTKLVASYLNEYLSFLPISEDENTALGYGEKGSKQHVVLSSGMITQ
C_coli_ICE1_Clade1a_Cas9      DTRWLTKLVASYLNEYLSFLPISEDENTALGYGEKGSKQHVVLSSGMITQ
C_coli_ICE1_Clade1c_Cas9      DTRWLTKLVASYLNEYLLFLPISEDENTALGYGEKGSKQHVVLSSGMITQ
C_coli_ICE1_Clade2_Cas9       DTRWLTKLVASYLNEYLLFLPISKDENTALGYGEKGSKQHVVLSSGMITQ
C_coli_ICE1_Clade3_Cas9       DTRWLTKLVASYLNEYLLFLPISEDENTALGYGEKGSKQHVVLSSGMITQ
                              ***::: *: .* ::** ***:...*:  *   :**** **   .**:*.
                                               RuvC III
C_jejuni_Cas9                 ALRHTWGFSAKDRNNHLHHAIDAVIIAYANNSIVKAFSDFKKEQESNSAE
C_coli_Clade1a_Cas9           ALRHTWGFSAKDRNNHLHHAIDAVIIAYANNSIVKAFSDFKKEQESNSAE
C_coli_Clade1b_Cas9           ALRHTWGFSAKDRNNHLHHAIDAAIIAYANNSIVKAFSDFKKEQESNSVE
C_coli_Clade1c_Cas9           VLRHTWGFAQKDRNNHLHHALDATIVAYSTNAIIKAFSDFKKEQELLKAK
```

```
C_coli_Clade2_Cas9          VLRHTWGFAQKDRNNHLHHALDATIVAYSTNAIIKAFSDFKKEQELLKAK
C_coli_Clade3_Cas9          VLRHTWGFAQKDRNNHLHHALDATIVAYSTNAIIKAFSDFKKEQELLKAK
C_jejuni_ICE1_Cas9          MLRNFWYLGFKNHKDYKNNAMDAIIVAFTTNSIIFAFNNFKKELDLAKAE
C_coli_ICE1_Clade1a_Cas9    MLRNFWYLGFKNHKNYKNNAMDAIIVAFTTNSIIFAFNNFKKELDLAKAE
C_coli_ICE1_Clade1c_Cas9    MLRNFWYLGFKNYKDYKNNAMDAIIVAFTTNSIIFAFNNFKKELDLAKAE
C_coli_ICE1_Clade2_Cas9     MLRNFWYLGFKNYKDYKNNAMDAIIVAFTTNSIIFAFNNFKKELDLAKAE
C_coli_ICE1_Clade3_Cas9     MLRNFWYLGFKNYKDYKNNAMDAIIVAFTTNSIIFAFNNFKKELDLAKAE
                             **: * :. *: ::: ::*:** *:*::.*:*: **.:**** :  ..:

C_jejuni_Cas9               LYAKKISELDYKNKRKFFEPFSGFRQKVLDKIDEIFVSKPERKKPSGALH
C_coli_Clade1a_Cas9         LYAKKISELDYKNKRKFFEPFSGFRQKVLDKIDEIFVSKPERKKPSGALH
C_coli_Clade1b_Cas9         LYAKKISELDYKNKRKFFEPFSGFRQKVLDKIDEIFVSKPERKKPSGALH
C_coli_Clade1c_Cas9         LYAKELTSDSYKHQAKFFEPFEGFREQILNQINKLFVSKPPRKRARGALH
C_coli_Clade2_Cas9          LYAKELTNDSYKHQAKFFEPFEGFREQILNQINKLFVSKPPRKRARGALH
C_coli_Clade3_Cas9          LYAKELTSDSYKHQAKFFEPFEGFREQILIQINKLFVSKPPRKRARGALH
C_jejuni_ICE1_Cas9          FYANKISESDYLLKRKFLPPFSGFKEQALEKVKNIFVSHSLKIKNKGTLH
C_coli_ICE1_Clade1a_Cas9    FYANKISESDYLLKRKFLPPFSGFKEQALEKVKNIFVSHSLKIKNKGTLH
C_coli_ICE1_Clade1c_Cas9    FYANKISESDYLLKRKFLPPFKGFKEQAFEKVNNIFVSHSLRIKNKGALH
C_coli_ICE1_Clade2_Cas9     FYANKISESDYLLKRKFLPPFKGFKEQAFEKVNNIFVSHSLRIKNKGALH
C_coli_ICE1_Clade3_Cas9     FYANKISESDYLLKRKFLPPFKGFKEQAFEKVNNIFVSHSLRIKNKGALH
                            :**::::. .*  : **: **.**::: : ::.::***:. : :  *:**

C_jejuni_Cas9               EETFRKEEEFYQSYGGKEGVLKALELGKIRKVNGKIVKN--GDMFRVDIF
C_coli_Clade1a_Cas9         EETFRKEEEFYQSYGGKEGVLKALELGKIRKVNGKIVKN--GDMFRVDIF
C_coli_Clade1b_Cas9         EETFRKEEEFYQSYGGKEGVLKALELGKIRKVNGKIVKN--GDMFRVDIF
C_coli_Clade1c_Cas9         KETFYSKDEMIKKYNSQEGVEIALNCGKIRKIGTKYVEN--DTMVRIDIF
C_coli_Clade2_Cas9          KETFYSKDEMIKKYNSQEGVEIALNCGKIRKIGTKYVEN--DTMVRIDIF
C_coli_Clade3_Cas9          KETFYPKDEMIKKYNSQEGLEIALNCGKIRKIGTKYVEN--DTIVRIDIF
C_jejuni_ICE1_Cas9          ELTPLKIKELKNTYG---DLDLAVKLGKIRKYNDKYYANANGSLVRADLF
C_coli_ICE1_Clade1a_Cas9    ELTPLKIKELKNTYG---DLDLAVKLGKIRKYNDKYYVNAKGSLARTDLF
C_coli_ICE1_Clade1c_Cas9    NLNPLKIKELKNTYK---DLEFAIASGRIRNYNGKFYSNANGSLVRVDLF
C_coli_ICE1_Clade2_Cas9     NLNPLKIKELKNTYK---DLEFAIASGRIRNYNGKFYSNANGSLVRVDLF
C_coli_ICE1_Clade3_Cas9     NLNPLKIKELKNTYK---DLEFAIASGRIRNYNGKFYSNANGSLVRVDLF
                            : .    .*: :.*    .:  *:  *:**: . *   *  . : * *:*

C_jejuni_Cas9               KHKKTNKFYAVPIYTMDFALKVLPNKAV--ARSKKGEIKDWILMDENYEF
C_coli_Clade1a_Cas9         KHKKTNKFYAVPIYTMDFALKVLPNKAV--ARSKKGEIKDWILMDENYEF
C_coli_Clade1b_Cas9         KHKKTNKFYAVPIYTMDFALKVLPNKAV--ARSKKGEIKDWILMDENYEF
C_coli_Clade1c_Cas9         KKQ--NKFYAIPIYTMDFALGVLPNKIVIAGKDKKGNPKQWQEIDESYEF
C_coli_Clade2_Cas9          KKQ--NKFYAIPIYTMDFALGVLPNKIVIIGKDKKGNPKQWQEIDESYEF
C_coli_Clade3_Cas9          KKQ--NKIYAIPIYTMDFALGVLPNKIVIKGKDKKGNPKQWQEIDESYEF
C_jejuni_ICE1_Cas9          VDK-KNKFHAVSIYKADFSTKKLPNKTP--ATTSNGETKEGIEMNENYNF
C_coli_ICE1_Clade1a_Cas9    VDK-ENKFHAVSIYKADFSTKKLPNKTP--ATTSNGETKEGIEMNENYNF
C_coli_ICE1_Clade1c_Cas9    VDK-KSKFHAVPIYKADFSTKQLPNKTP--AAISNGKIKEGVEMNENYNF
C_coli_ICE1_Clade2_Cas9     VDK-KSKFHAVPIYKADFSTKQLPNKTP--AAISNGKIKEGVEMNENYNF
C_coli_ICE1_Clade3_Cas9     VDK-KSKFHAVPIYKADFSTKQLPNKTP--AAISNGKIKEGVEMNENYNF
                             .:  .*::*:.**. **:   ****    .  .:*: *:   ::*.*:*

C_jejuni_Cas9               CFSLYKDSLILIQTKDMQEPEFVYYNAFTSSTVSLIVSKHDNKFETLSKN
C_coli_Clade1a_Cas9         CFSLYKDSLILIQTKDMQEPEFVYYNAFTSSTVSLIVSKHDNKFETLSKN
C_coli_Clade1b_Cas9         CFSLYKDSLILIQTKDMQEPELVYFNAFTSSTVSLIVSKHDNKFETLSKN
C_coli_Clade1c_Cas9         CFSLHKDDLVLIQKKDMQEPEFAYYNGFDISNSSICVEKHDNKFENLTDN
C_coli_Clade2_Cas9          CFSLHKDDLVLIQKKDMQEPEFAYYNGFDISNSSICVEKHDNKFENLTDN
C_coli_Clade3_Cas9          CFSLHKDDLVLIQKKDMQEPEFAYYNSFDISNSSICVEKHDNKFENLTDN
C_jejuni_ICE1_Cas9          CMSLYKNTPISVKIKGMKEPIICYYHGFNTSGSKITYKKHDNNYHNLSED
C_coli_ICE1_Clade1a_Cas9    CMSLYKNTPVSVKIKGMKEPIICYYHGFNTSGSKITYKKHDNNYHNLSED
C_coli_ICE1_Clade1c_Cas9    YISLYKNTPISVKIKGMEEPIICYYHGFNISGSQIIYKKHDNNYYNLSKD
C_coli_ICE1_Clade2_Cas9     YISLYKNTPISVKIKGMEEPIICYYHGFNISGSQIIYKKHDNNYYNLSKD
C_coli_ICE1_Clade3_Cas9     YISLYKNTPISVKIKGMEEPIICYYHGFNISGSQIIYKKHDNNYYNLSKD
                             :**:*:  : :: *.*:** : *::.*  *  .:  .****:: .*:.:

C_jejuni_Cas9               QKILFKNANEKEVIAKSIGIQNLKVFEKYIVSALGEVTKAEFRQREDFKK
C_coli_Clade1a_Cas9         QKILFKNANEKEVIAKSIGIQNLKVFEKYIVSALGEVTKAEFRQREDFKK
C_coli_Clade1b_Cas9         QKILFKNANEKEVIAKSIGIQNLKVFEKYIVSALGEVTKAEFRQREDFKK
C_coli_Clade1c_Cas9         QKLLFVNAKEGSVKAEKLGIQGLKIFEKYIITPLGEKIKADFKPREDIAL
C_coli_Clade2_Cas9          QKLLFTNAEEGNVKAKKIGIQGLRIFEKYIITPLGEKIKADFKPREDIAL
C_coli_Clade3_Cas9          QKLLFTNAEEGNVKAKKLGIQGLKIFEKYIITPLGEKIKADFKLREDIAL
C_jejuni_ICE1_Cas9          EMVVFRKNDKE-----SIAVGKILEIKKYSISPSGELSLIENEERKWF--
C_coli_ICE1_Clade1a_Cas9    EMVVFRKNDKE-----SIVVGKILEIKKYSISPSGELSLIENEKRKWF--
C_coli_ICE1_Clade1c_Cas9    EMAIFRKGDKE-----AIAIGRMLEIKKYNISPSGELILIENEERKWF--
C_coli_ICE1_Clade2_Cas9     EMAIFRKGDKE-----AIAIGRMLEIKKYNISPSGELILIENEERKWF--
```

```
C_coli_ICE1_Clade3_Cas9       EMAIFRKGDKE-----AIAIGRILEIKKYNISPSGELILIENEERKWF--
                              :  :* : .:       : :  :  ::** ::. **    : . *: :

C_jejuni_Cas9                 --------
C_coli_Clade1a_Cas9           --------
C_coli_Clade1b_Cas9           --------
C_coli_Clade1c_Cas9           KTSKKHGL
C_coli_Clade2_Cas9            KTSKKHGI
C_coli_Clade3_Cas9            KTSKKHGL
C_jejuni_ICE1_Cas9            --------
C_coli_ICE1_Clade1a_Cas9      --------
C_coli_ICE1_Clade1c_Cas9      --------
C_coli_ICE1_Clade2_Cas9       --------
C_coli_ICE1_Clade3_Cas9       --------
```

ICE1-like elements in *Campylobacter*

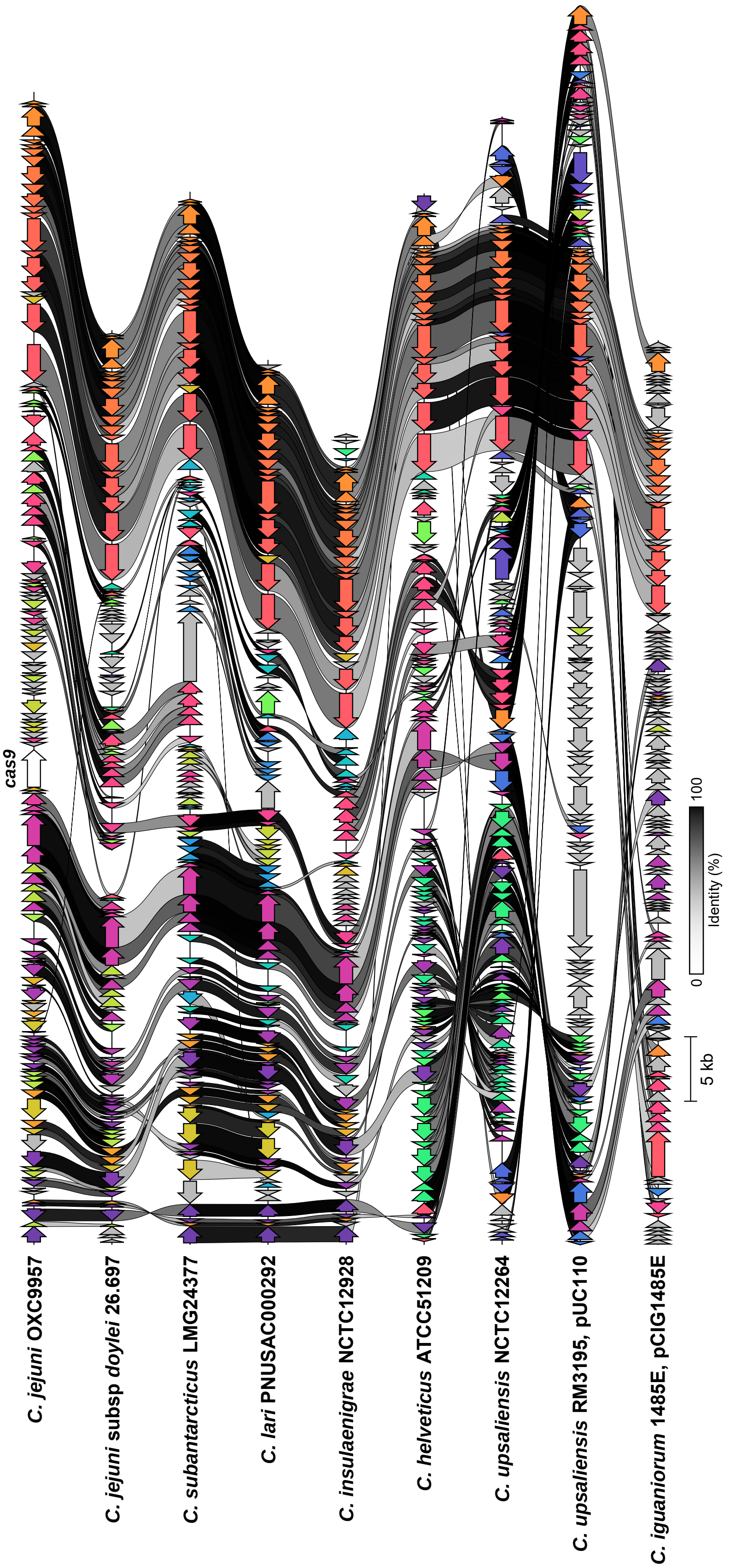

Van Vliet et al, Figure S2

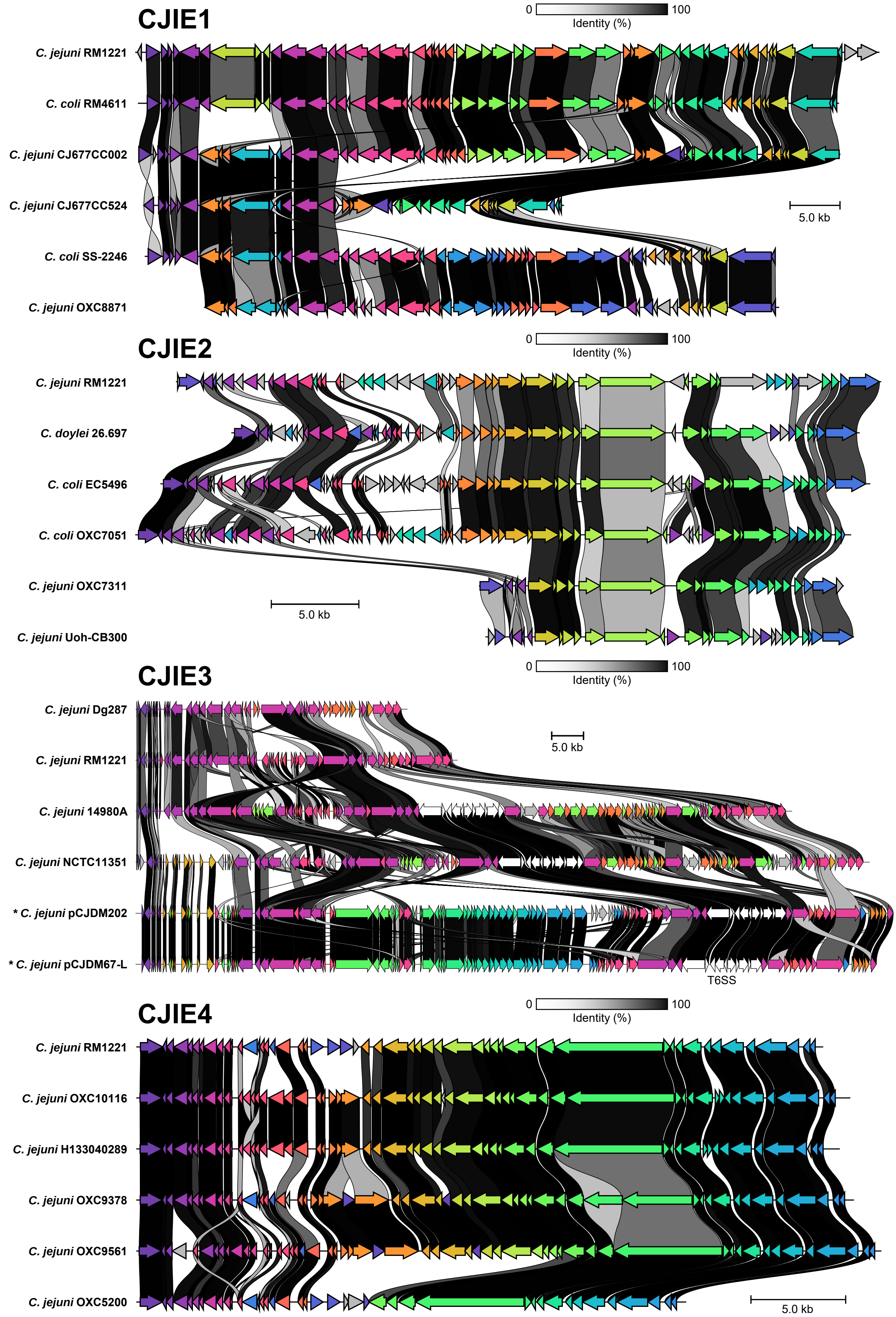

Van Vliet et al, Figure S3

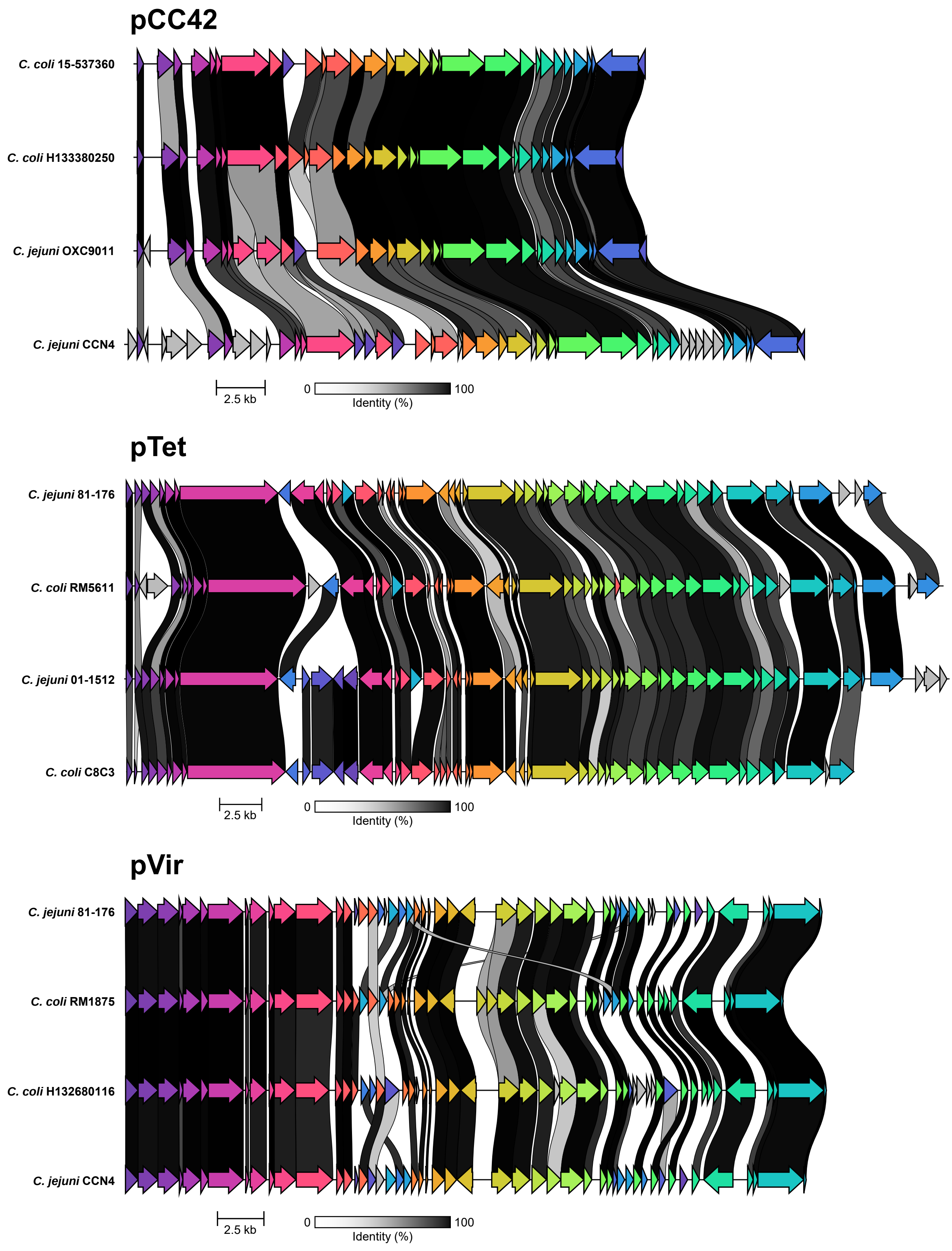

Van Vliet et al, Figure S4

Table S4. Distribution of CampyICE1 plasmid-specific CRISPR-spacers and pVir, pTet and pCC42 plasmids in CampyICE1-positive *C. jejuni* and *C. coli*.

**plasmid**	**no plasmid**		**plasmid present**		
**spacer** [a]	**absent**	**matched** [b]	**absent**	**ΔCas9+mismatch**[b]	**matched** [b]
***C. jejuni* (n=133)**					
pVir	115	16	2	0+0	0
pTet	11	105	2	2+5	8
pCC42	22	97	3	0+4	7
***C. coli* (n=81)**					
pVir	27	50	3	1+0	0
pTet	8	58	3	1+11	0
pCC42	2	57	0	2+9	11

a. Plasmid spacers identified by CRISPRfinder, CRISPR Recognition Tool CRT and manual searches were screened for matches with Campylobacter plasmids using CRISPRtarget.

b. CampyICE1-positive genomes positive for pVir, pTet and pCC42 were searched with the plasmid-targeting spacers using BLAST, and recorded for perfect matches and imperfect matches. This was to allow for possible sequence differences with the reference pVir, pTet and pCC42 plasmid sequences, or alternatively to detect mutations introduced to escape CRISPR-Cas functionality. In addition, the presence of a full-length *cas9* gene was checked, as this is required for CRISPR-Cas9 functionality.
